# Expression of extra-cellular levansucrase in *Pseudomonas* syringae is controlled by the *in planta* fitness-promoting metabolic repressor HexR

**DOI:** 10.1186/s12866-015-0349-0

**Published:** 2015-02-26

**Authors:** Amna Mehmood, Khaled Abdallah, Shaunak Khandekar, Daria Zhurina, Abhishek Srivastava, Nehaya Al-Karablieh, Gabriela Alfaro-Espinoza, Daniel Pletzer, Matthias S Ullrich

**Affiliations:** Molecular Life Science Research Center, Jacobs University Bremen, Campus Ring 1, Bremen, 28759 Germany; Hamdi Mango Center for Scientific Research, The University of Jordan, P.O. Box 13507, Amman, 11942 Jordan

**Keywords:** Plant pathogen, Bacterial blight, Soybean, *Pseudomonas syringae*, Levansucrase, Hexose metabolism, HexR

## Abstract

**Background:**

*Pseudomonas syringae* pv. glycinea PG4180 causes bacterial blight on soybean plants and enters the leaf tissue through stomata or open wounds, where it encounters a sucrose-rich milieu. Sucrose is utilized by invading bacteria via the secreted enzyme, levansucrase (Lsc), liberating glucose and forming the polyfructan levan. *P. syringae* PG4180 possesses two functional *lsc* alleles transcribed at virulence-promoting low temperatures.

**Results:**

We hypothesized that transcription of *lsc* is controlled by the hexose metabolism repressor, HexR, since potential HexR binding sites were identified upstream of both *lsc* genes. A *hexR* mutant of PG4180 was significantly growth-impaired when incubated with sucrose or glucose as sole carbon source, but exhibited wild type growth when arabinose was provided. Analyses of *lsc* expression resulted in higher transcript and protein levels in the *hexR* mutant as compared to the wild type. The *hexR* mutant’s ability to multiply *in planta* was reduced. HexR did not seem to impact *hrp* gene expression as evidenced by the *hexR* mutant’s unaltered hypersensitive response in tobacco and its unmodified protein secretion pattern as compared to the wild type under *hrp*-inducing conditions.

**Conclusions:**

Our data suggested a co-regulation of genes involved in extra-cellular sugar acquisition with those involved in intra-cellular energy-providing metabolic pathways in *P. syringae*.

**Electronic supplementary material:**

The online version of this article (doi:10.1186/s12866-015-0349-0) contains supplementary material, which is available to authorized users.

## Background

Fructan or glucan polymers are formed wherever microbes encounter sucrose-rich conditions, would it be in association with plants, in the oral cavity, in food manufacturing, or during bio-fuel production processes [1]. When plant-borne sucrose is present, the soybean-infecting bacterial blight pathogen, *Pseudomonas syringae* pv. glycinea, uses levansucrase (EC 2.4.1.10, Lsc) to synthesize the extra-cellular high-molecular fructofuranan, levan, thereby releasing glucose for primary metabolism. Three levansucrase-encoding genes, *lscA*, *lscB*, and *lscC*, were identified in *P. syringae* pv. glycinea PG4180, from which only *lscB* and *lscC* are expressed, as a mutant lacking *lscB* and *lscC* but possessing *lscA* is levan-deficient [2]. Furthermore quantitative expression analysis of *lsc* genes by quantitative Reverse Transcriptase (qRT)-PCR showed that *lscB* and *lscC* are actively expressed. However *lscA* is not being expressed due to an altered upstream region of *lscA* which does not seem to promote *lsc* expression [3]. Both enzymes are synthesized maximally at 18°C *in vitro* and *in planta* and their expression is optimal at the early logarithmic growth stage [4,5].

Bacterial communities growing epiphytically on plants are primarily affected by carbon availability as supported by the finding that very low sugar concentrations are sufficient to support the growth of 10^7^ to 10^8^ cells per leaf [6]. Stomatal openings and wounds provide the site of entry for *P. syringae*. Under favorable micro-environmental conditions, the bacterial cells live endophytically and subsequently initiate the infection process via production of the phytotoxin coronatine [7,8] and attachment to plant cell surfaces. The infection process is fostered by low environmental temperatures such as 18-20°C as opposed to the optimal growth temperature of *P. syringae*, 28°C [9,10]. A complex sequence of events mediated by injection of bacterial hypersensitive reaction and pathogenicity (Hrp) effector proteins into plant cells [11] ultimately activates plant-borne K^+^ efflux and H^+^ influx, which increases the apoplastic pH from 5.5 to 7.5 [12]. Subsequently, this high extra-cellular pH induces efflux of the dominant photo assimilate, sucrose, from plant cells [12]. Apoplastic sucrose ranging in concentrations from 20 μM to 1–5 mM is hydrolyzed by either plant-borne invertases or by extra-cellular microbial enzymes, e.g. Lsc [13,14].

For glucose metabolism, metabolic pathway structures vary among bacterial species with different ecological niches [15]. In contrast to enterobacteria [16], pseudomonads utilize the Entner-Doudoroff (ED) pathway due to lack of 6-phosphofructokinase and hence do not catabolize sugars via the Embden-Meyerhof-Parnas pathway [17,18]. The ED pathway can be linear, alternative, modified with non-phosphorylated intermediates, or cyclic [19] and was first described in *Pseudomonas saccharophila*, which now belongs to the order *Burkholderiales* [17,20].

The genes required for glucose metabolism in *Pseudomonas putida* KT2440 are organized in several operons [21]. The first operon consists of the *zwf*, *pgl*, and *eda* genes coding for glucose-6-phosphate dehydrogenase, 6-phosphogluconolactonase (both enzymes of the glucose phosphorylative pathway), and Eda (an enzyme of the Entner-Doudoroff pathway) [22,23]. Divergently directed to this operon is the gene encoding for the hexose metabolic repressor, hexR [21]. The second operon consists of the *edd* and *glk* genes that encode 6-phosphogluconate dehydratase (the first enzyme of the Entner-Doudoroff pathway) and glucokinase (an enzyme of the glucose phosphorylative pathway), respectively [22,23]. Divergently directed to this operon is the gene *gap1* which codes for glyceraldehydes 3-phosphoste dehydrogenase [21]. The transcription of these genes and operons is negatively regulated by HexR. Two monomers of HexR bind to the promoter regions of *edd*, *zwf*, and *gap-1* genes by recognizing a palindromic sequence TTGTN7–8ACAA [21,23-25]. In the current study, it was hypothesized that in *P. syringae* not only genes involved in cellular glucose metabolism but also genes encoding extra-cellular Lsc were controlled by HexR. In turn, this might have consequences for our understanding about what determines bacterial *in planta* fitness and potentially virulence.

In order to address this hypothesis, a *hexR* mutant was generated in PG4180 and tested for its growth in minimal medium containing glucose, sucrose, or arabinose as sole carbon source. Analyses of *lsc* expression by qRT-PCR and Western blotting were conducted for the PG4180 wild type and its *hexR* mutant. The mutant was compared to the wild type in terms of its *in planta* fitness. Furthermore, hypersensitive response (HR) reactions and profiles of secreted proteins were compared for the wild type and the *hexR* mutant when grown under *hrp*-inducing conditions.

## Methods

### Bacterial strains, plasmids and growth conditions

Bacterial strains and plasmids used in this study are listed in Table 1. *Escherichia coli* was maintained at 37°C on Luria-Bertani (LB) medium [26]. *P. syringae* was routinely maintained at 28°C on mannitol-glutamate (MG) medium [27]. For liquid cultures at 18°C, bacteria were grown in 200 ml of Hoitink-Sinden (HS) medium [28] supplemented with various carbon sources in 1-l Erlenmeyer flasks. 113 mM of glucose (HS + glucose) was replaced by 57 mM of sucrose in HS + sucrose medium while HS + arabinose medium had the following constituents: 0.8 mM MgSO_4_, 30 mM KH_2_PO_4_, 16 mM K_2_HPO_4_, 16 mM KNO_3_, 20 μM FeCl_3_, 133 mM L-arabinose. Bacterial growth was continuously monitored by measuring the optical density at 600 nm (OD_600_). Antibiotics were added to media at the following concentrations (μg/ml): ampicillin, 50; spectinomycin, 25; kanamycin, 25; tetracycline, 25; gentamicin, 2.

**Table 1:** **Bacterial strains and plasmids used in this study**

**Strain**	**Relevant characteristics** ^**a**^	**Reference/source**
*Escherichia coli*		
DH5α	*supE44 ΔlacU169 (Φ80 lacZΔM15) hsdR17 recA1 endA1 gyrA96 thi-1 relA1*	[26]
*Pseudomonas syringae* pv. Glycinea		
PG4180	wild type, levan+	[29]
*hexR*	Km^r^, *hexR* mutant of PG4180, levan++	This study
**Plasmid**		
pRK2013	Km^r^, helper plasmid	[30]
pBBR1MCS	Cm^r^, broad-host-range cloning vector	[31]
pBBR1MCS-3	Tc^r^, broad-host-range cloning vector	[32]
pGEM-T Easy	Ap^r^, vector for cloning of PCR products	Promega
pEX18Ap	Ap^r^, *oriT*+ *sacB*+ gene replacement vector	[33]
pFKm	Source of Km cassette flanked with FRT sequences	[34]
pGEM.hexR1	Ap^r^, contains 456-bp upstream region of *hexR*	This study
pGEM.hexR2	Ap^r^, contains 360-bp downstream region of *hexR*	This study
pGEM.hexR1-Km	Ap^r^, Km, 1230-bp *Kpn*I fragment containing Km-FRT cassette of pFKm1 cloned into pGEM.hexR1	This study
pGEM.hexR-Km	Ap^r^, Km, 360-bp *Bam*HI*-Spe*I fragment of pGEM.hexR2 cloned into pGEM.hexR1-Km	This study
pEX.hexR-Km	Ap^r^, Km, 2046-bp *Eco*RI fragment of pGEM.hexR-Km cloned into pEX18Ap	This study

^a^Ap, ampicillin; Cm, chloramphenicol; Gm, gentamicin; Km, kanamycin; Sp, spectinomycin; Tc, tetracycline.

### Generation of *hexR* mutant in PG4180

A *P. syringae* pv. glycinea PG4180 *hexR* mutant was generated using the broad-host-range Flp-FRT recombination system [33]. Two fragments flanking the *hexR* gene were amplified from PG4180 genomic DNA using two pairs of primers: HexR_1f/HexR_1r and Hex_2f/HexR_2r (Table 2). PCR products were cloned into pGEM-T Easy (Promega) yielding plasmids pGEM.HexR1 and pGEM.HexR2 (Table 1). A 1,230-bp *Kpn*I fragment containing a Km^R^ cassette flanked with FRT sequences was removed from plasmid pFKm [34] and ligated into *Kpn*I-digested pGEM.HexR1, yielding pGEM.HexR1-Km. A 360-bp *Spe*I-*Bam*HI fragment digested from pGEM.HexR2 was ligated into *Spe*I-*Bam*HI-digested pGEM.HexR1-Km, yielding plasmid pGEM.HexR-Km. Finally, a 2,046-bp *Eco*RI fragment was removed from pGEM.HexR-Km and ligated into *Eco*RI-digested plasmid pEX18Ap [33], yielding the *hexR* gene replacement plasmid pEX.HexR-Km. This plasmid was mobilized into *P. syringae* PG4180 by tri-parental mating. Putative mutants were screened on MG medium agar plates supplemented with kanamycin and were subsequently confirmed for the genotype by PCR using primers hex_up_fwd and hex_down_rev (Table 2).

**Table 2:** **Oligonucleotide primers used in this study**

**Oligonucleotides**	**Nucleotide sequence (5′ to 3′)**
HR_1f^a^	*GGATCC* GTTCAACTCATCGAGTC
HR_1r	CAGATGCGACTGTTCGTC
HR_2f	GACCCCCGGATCAGTGCCAG
HR_2r^a^	*GGATCC GGTACC* CAGCCGCTATCCGATCGAG
lscB/C_RT_fwd	TCGGTTATCCTGACCCTGAC
lscB/C_RT_rev	CCATGACGATCTTCCCAGTC
lscB_hex_fwd	CGCAATTAATGCGAGCCCGCAGG
lscB_hex_rev	TTGCATTGGTCTCCTTGTGCTTC
hex_up_fwd	CGAGCAAGTCGCACCG
hex_down_rev	GAAGTCGACATGCAGGTAG
hexR_fwd	GAATTCATGGACCGCGTAAGAAACC
hexR_rev	CTGCAGTCAGCCTTGATCCTCGATC
hexR_verif_fwd	CTCAACCCGCAGATGGCAA
hexR_verif_rev	CGATGACCTCGCGGATCAT

^a^Restriction sites in the primers are in italics: GGATCC - *Bam*HI, GGTACC - *Kpn*I.

### Verification of the *hexR* mutant’s phenotype by Reverse-Transcriptase Polymerase Chain Reaction (RT-PCR)

Template-specific primers were designed for *hexR* and *lscB/C* genes of *P. syringae* PG4180. Bacterial cells were grown in HS + arabinose medium and harvested at an OD_600_ of 0.5. RNA was extracted by acid phenol/chloroform extraction method [5]. RT-PCR was performed on total RNA using RevertAid First Strand cDNA Synthesis Kit (Thermo Scientific, Schwerte, Germany). The hexR_fwd, hexR_rev, hexR-verif_fwd, hexR_verif_rev and lscB/C primers were used to check for presence of a *hexR* and *lscB/C* mRNA by PCR using cDNA as template. Regular PCR with the same primer-pairs and genomic DNA as template were used as control. The thermocycler program was as follows: 1 cycle of 95°C for 60 s; 30 cycles of 95°C for 10 s, 60°C for 30 s, 72°C for 30 s; 1 cycle of 72°C for 5 min. The results were analyzed by 1% agarose gel electrophoresis.

### Analysis of *lsc* expression by quantitative Reverse Transcriptase Polymerase Chain Reaction (qRT-PCR)

Bacterial cells were grown in HS + arabinose medium at 18°C. When cultures reached respective OD_600_ values, total RNA was isolated by acid phenol/chloroform extraction, and samples were normalized by multiplexed fluorescent Northern hybridization and 23S rRNA transcript amount comparison as described previously [5]. Yield and purity of RNA were determined by measuring absorption at 260 and 280 nm. Total RNA samples were treated with TURBO DNA-free (Applied Biosystems, Darmstadt, Germany) to remove remaining traces of genomic DNA as described by the manufacturer’s recommendation.

SYBR green-based qRT-PCR was performed with 1 ng normalized RNA template and 200 nM primers (lscBC_RT_fwd, lscBC_RT_rev) using the QuantiTect SYBR Green one-step RT-PCR Kit (Qiagen, Hilden, Germany) according to the manufacturer’s instructions. The thermocycler program comprised an initial step of 95°C for 15 min followed by 40 cycles of 95°C for 30 s, 55°C for 30 s, 72°C for 30 s. Reactions were performed in technical duplicates and biological triplicates with a Mastercycler® realplex2 real-time PCR system (Eppendorf, Hamburg, Germany) as described by the manufacturer using their universal program. Reactions with no addition of reverse transcriptase served as negative controls and proved lack of DNA contamination. Specificity of amplification was assessed by analyzing the melting curve of the amplification product. Due to very high sequence identity between *lscB* and *lscC* it was not possible to design primers discriminating between these two mRNAs, thereby expression profile of *lsc* is always referred as a combination of both genes.

### Immunological detection of Lsc

Generation and concentration of cell-free supernatants of *P. syringae* cells and the use of polyclonal antibodies were carried out as described previously [4]. Cultures were grown in HS+ arabinose medium. Equal aliquots of protein fractions were loaded (0.5, 2.5 and 5 µg/lane) and separated by 10% SDS-PAGE. Electrophoresis, electro-blotting on nitrocellulose membranes, and immunodetection were conducted by standard procedures [26].

### Growth of PG4180 wild type and *hexR* mutant *in planta*

*In planta* growth of PG4180 and its *hexR* mutant was evaluated on soybean plants. Soybean seedlings were germinated and grown in an environmentally controlled chamber for approximately three weeks prior to the growth assays. PG4180 wild type and *hexR* mutant were incubated for 48 h at 28°C on MG agar plates. Cells were suspended in distilled water, adjusted to an OD_600_ of 0.1 (corresponding to approximately 10^7^ CFU/ml) and applied to the leaves with an airbrush (~8 psi) until the leaf surfaces were uniformly wet. Subsequently, humid environment was achieved by enclosing the inoculated plants with a clear plastic bag for overnight. Inoculated plants were grown in a plant growth chamber (19-21°C) with a 12-hr light period. At days 1, 3, 5, 7, 9, 11 and 14 after inoculations, two individual leaves were randomly excised from plants corresponding to each inoculums and their weight was measured. Epiphytic bacteria were isolated by placing leaves in a 50-ml falcon tubes containing 20 ml of external wash buffer (0.1 M potassium phosphate, 0.1% bactopeptone and pH = 7.0) and the tubes were sonicated for 7 minutes [35]. Leaves were then removed and macerated in 20 ml of isotonic solution (0.9% NaCl) using sterile mortar and pestle. Bacterial counts (CFU/g fresh weight) were determined by plating dilutions from external wash buffer and leaf homogenate onto MG agar and counting of fluorescent colonies after incubation at 28°C for 96 h.

### Hypersensitive response assay on tobacco

Tobacco plants (*Nicotiana tabacum* cv. Petit Havana SR1) were grown in a light chamber at 20 to 25°C, 60% humidity, with a 12-h photoperiod (15,000 lux). PG4180 wild type and *hexR* mutant were incubated for 48 h at 28°C on MG agar. Cells were suspended in sterile 0.9% NaCl, adjusted to an OD_600_ of 0.1 (corresponding to approximately 10^7^ CFU/ml). Tobacco plants were inoculated with bacterial suspensions by syringe injection of leaf veins of the third and fourth leaf. As negative control sterile 0.9% NaCl was used. Infiltrated areas were monitored for development of the hypersensitive reaction in form of necrosis after 24 and 48 h.

### Extra-cellular protein pool preparation and SDS-PAGE

For sample preparation of secreted proteins, bacteria were grown in two consecutive overnight pre-cultures in King’s B broth [36] at 28°C. From the first pre-culture, the cell suspension was adjusted to an OD_600_ of 0.1 with 50 ml of fresh King’s B broth and incubated at 28°C. Bacterial cultures were harvested at an OD_600_ of 1.0 and centrifuged at 4,000 rpm for 30 min. The bacterial cells were washed two times with Hrp-inducing medium [37,38] or with HS+ glucose medium [28], respectively. Subsequently, the cell pellets were resusp ended in 50 ml of Hrp-inducing medium or HS+ glucose medium at 28°C and incubated shaking at 250 rpm for 4 h. Next, 25 ml of the bacterial cultures were centrifuged at 4,000 rpm for 30 min. Cell-free supernatant samples were prepared by filter-sterilization through 0.2-μm pore size membrane filters and concentrated 50-fold using 10-kDa millipore filters (Amicon, Billerica, USA). Extra-cellular protein samples were stabilized in 50 mM Tris–HCl (pH 8.8). Total protein amounts were determined using a Nanodrop apparatus (Thermo Fisher Scientific, Langenselbold, Germany), 10 μl of protein samples were separated by 12.5% SDS-PAGE and subsequently visualized by silver staining according to established procedures [39].

## Results

### Generation and genotypic characterization of the *P. syringae hexR* mutant

T del Castillo, E Duque and JL Ramos [23] and A Daddaoua, T Krell and JL Ramos [22] had previously shown that genes encoding enzymes of the phosphorylative branch and the ED pathway of glucose catabolism in *P. putida* were regulated by the hexose metabolism repressor, HexR. In *P. syringae*, extra-cellular Lsc releases glucose by sucrose hydrolysis. In order to investigate the impact of HexR on expression of *lscB* and *lscC* genes, a *hexR*-deficient mutant of *P. syringae* PG4180 was generated by insertion of a kanamycin resistance cassette in the *hexR* gene as a result of homologous recombination. The resulting mutant genotype was verified by growth on kanamycin-containing medium and RT-PCR analysis (Additional file 1: Figure S1).

### *In silico* analysis of HexR binding site upstream of *lsc* genes

**Figure 1:**
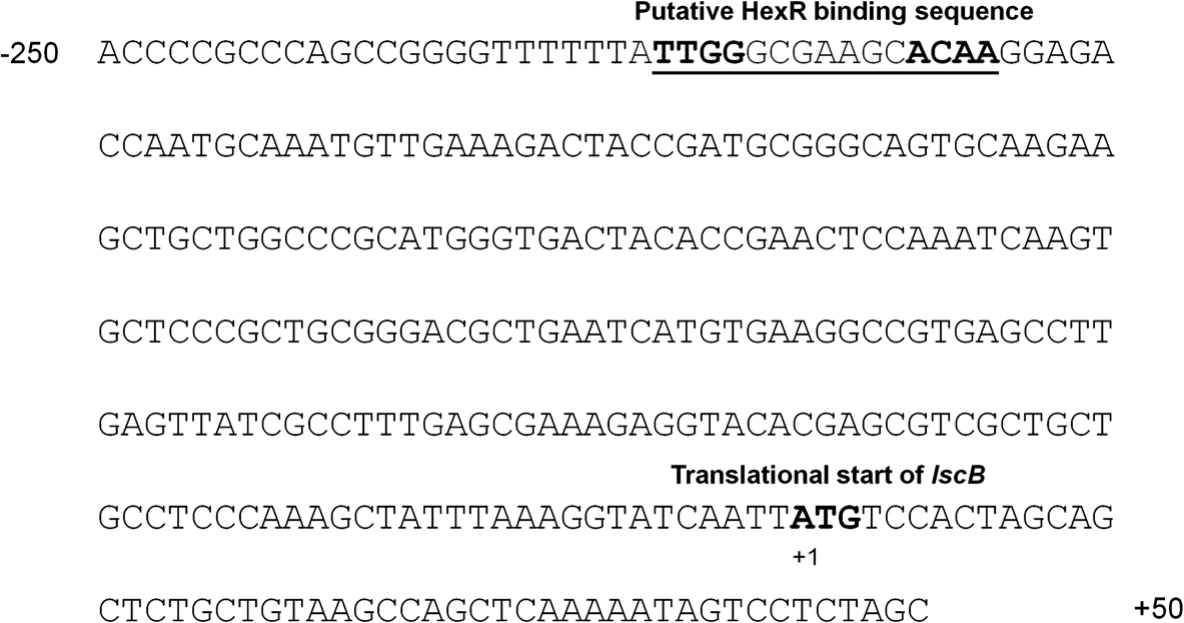
**Nucleotide sequence of the upstream region of** ***lscB*** **.** Sequence contains a putative HexR binding site (underlined) similar to that described by A Daddaoua, T Krell and JL Ramos [22]. +1 represents the translational start site of *lscB*.

Analysis of the nucleotide sequences upstream of *lscB* and *lscC*, which are almost identical at nucleotide level [2,40], revealed the presence of a sequence (nucleotides +113 to +127 with respect to the corresponding translational start site) with high similarity to the conserved motif TTGTN_7–8_ACAA previously shown to be the DNA binding site of HexR in *P. putida* [22-24] (Figure 1). This finding suggested a putative binding of HexR of *P. syringae* to the upstream sequence of both *lsc* genes.

### Comparison of different HexR protein sequences

Multiple sequence alignments of several HexR protein sequences derived from Lsc-producing *P. syringae* strains revealed that those proteins have a high pair-wise identity scores of >97% (data not shown). PFAM analysis [41] of these proteins showed that there is a DNA binding domain (PFAM01418) and a sugar isomerase (SIS) binding domain (PFAM 01380) as described for *P. putida* KT2440 by A Daddaoua, T Krell and JL Ramos [22] (Additional file 2: Figure S2) suggesting a similar way of enzymatic activity. A consensus sequence obtained from multiple alignments of HexR sequences from several *P. syringae* strains revealed 91% identity at the amino acid sequence level to that of *P. putida* (Additional file 3: Figure S3).

### *In vitro* and *in planta* growth of *P. syringae* PG4180 and its *hexR* mutant

**Figure 2:**
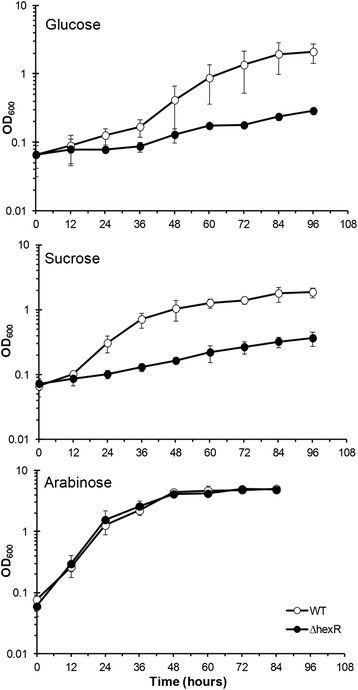
**Growth curve of PG4180 wild type (WT) (o) and its** ***hexR*** **mutant (●).** Cultures are grown in HS minimal media supplemented with glucose, sucrose, or arabinose as sole carbon source at 18°C. Error bars represent standard deviation of the mean of three biological replicates (n = 3).

To compare the growth of PG4180 wild type and its *hexR* mutant were grown in minimal media containing different carbon sources at 18°C (Figure 2). In contrast to the wild type, the *hexR* mutant did not grow significantly in HS medium containing 20 g/ml (113 mM) of glucose. Replacing glucose by 10 g/ml (57 mM) of sucrose, thereby providing an equal total number of carbon atoms did not change the weak growth phenotype of the *hexR* mutant. However, growth of the wild type was unaffected by this change of carbon source.

When glucose was replaced by an equal amount of 20 g/ml (133 mM) arabinose as the sole carbon source, growth of the *hexR* mutant was not distinguishable from that of the wild type indicating that HexR might not be involved in regulation of pathways utilizing arabinose, and that the *hexR* mutant growth phenotype was restricted to glucose utilization (Figure 2). For the wild type, sucrose apparently allowed for a faster adaptation as sucrose-supplemented cultures grew faster during early logarithmic growth. No significant difference was observed for the *hexR* mutant grown on glucose- or sucrose-supplemented medium. Arabinose allowed for the most efficient growth independent of the genotypes studied. These results indicated a potential HexR-mediated regulatory link of intra-cellular hexose metabolism with synthesis of Lsc since sucrose is the source of glucose via the enzymatic activity of the later enzyme.

**Figure 3:**
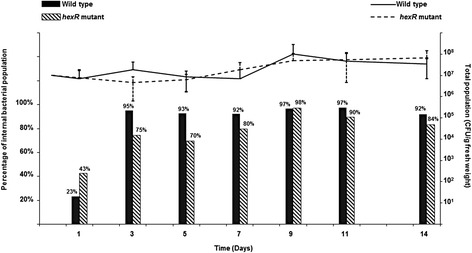
**Total population of PG4180 wild type (**  **) and its hexR mutant (**  **) in soybean leaves.** Columns represent the percentage of internalized PG4180 wild type (Black) and its *hexR* mutant (Black stripes). Bacterial suspensions were spray-inoculated on leaves of soybean plants grown in a greenhouse at 19-21°C. Data represent the mean values from five independent experiments with each two leaf samples. Error bars represent standard deviation of the mean of five biological replicates (n = 5).

To analyze the effect of a *hexR* mutation on growth *in planta*, PG4180 wild type and its *hexR* mutant were spray-inoculated onto soybean leaves with suspensions adjusted to 1 × 10^7^ CFU/ml. Subsequently, the plants were kept in a plant growth chamber and bacteria were recovered between days 1 to 14 post inoculation (Figure 3). Results showed that the total population number and percentage of internalized bacteria were not significantly different between plants inoculated with PG4180 wild type or its *hexR* mutant. However, a clear trend was observed for the percentage of bacteria that entered the interior of the leaf tissue. Except for days 1 and 9, plants inoculated with PG4180 wild type showed a higher percentage of internalized bacterial population when compared to plants subjected to the *hexR* mutant. These results might indicate an important role of HexR for the *in planta* fitness of *P. syringae*.

### Transcriptional analyses of *lsc* genes

**Figure 4:**
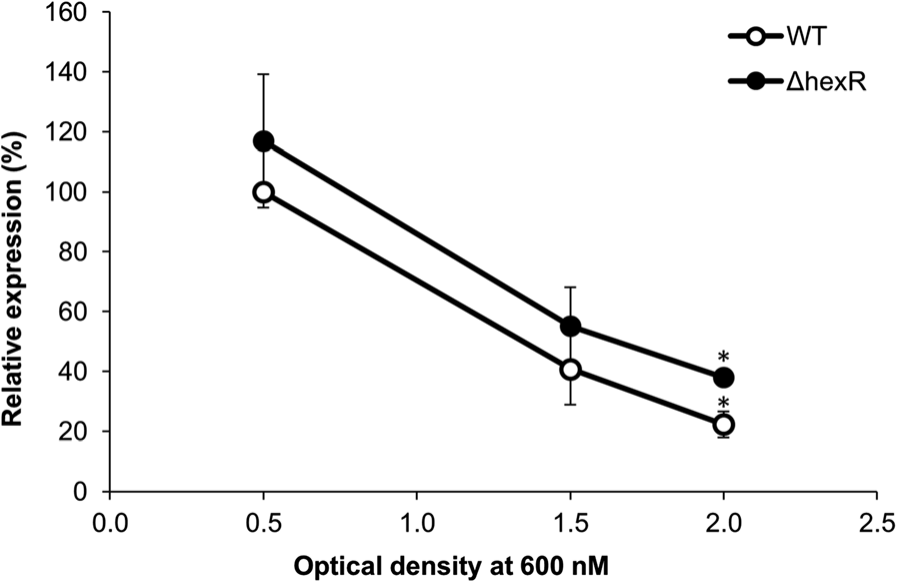
**Quantitative Reverse Transcriptase PCR analysis of growth phase-dependent** ***lsc*** **gene expression.** PG4180 and its *hexR* mutant were grown at 18°C in HS + arabinose. Relative mRNA levels were related to the mean value determined for the signals of PG4180 wild type at an OD_600_ of 0.5, which was defined as 100%. Data show the means and standard errors of three biological replicates (n = 3) (* = P < 0,005).

To analyze the effect of the *hexR* mutation on *lsc* gene expression, a growth-phase dependent transcriptional analysis was conducted using qRT-PCR. Cells of PG4180 wild type and the *hexR* mutant were grown in minimal medium containing arabinose at 18°C since levan production was shown to be maximal at this temperature [4]. Transcription of *lsc* genes in wild type and *hexR* mutant was highest during the early exponential growth phase and significantly decreased during further growth (Figure 4). Expression of *lsc* in the *hexR* mutant showed a tendency of being higher than the wild type in the early and mid-logarithmic growth phase. Interestingly, a significantly higher expression (p < 0.01) of *lsc* was observed in the *hexR* mutant as compared to that of the wild type at an OD_600_ of 2.0 referring to late-logarithmic to stationary phase.

### Abundance of Lsc in PG4180 wild type and *hexR* mutant

**Figure 5:**
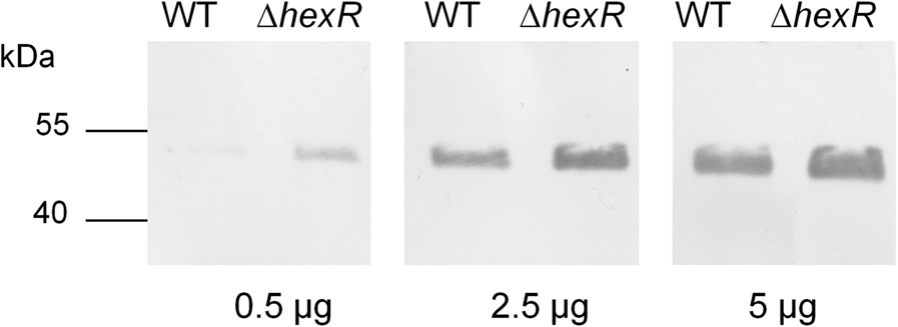
**Qualitative Western blot analysis of extra-cellular Lsc in cell-free supernatant of PG4180 wild type (WT) and its** ***hexR*** **mutant.** Cultures are grown in HS medium supplemented with arabinose as sole carbon source at 18°C. 0.5, 2.5, 5 μg of protein samples per lane were electrophoretically separated, transferred to a polyvinylidene fluoride membrane, and hybridized with Lsc-specific polyclonal antibodies.

To qualitatively assess levan formation, PG4180 wild type and its *hexR* mutant were grown on sucrose-containing MG agar plates resulting in indistinguishable levels of levan formation for both (Data not shown). The accumulation of Lsc in extracellular fractions of the wild type and the *hexR* mutant of PG4180 was tested using immunological detection. Protein samples were obtained from cell-free culture supernatants of bacterial cultures grown with arabinose as sole carbon source to late exponential phase at 18°C. Comparison of wild type and *hexR* mutant showed a slightly higher Lsc accumulation in the mutant’s culture supernatant (Figure 5). These results further supported the hypothesis that HexR might repress *lsc* gene expression, resulting in more extra-cellular accumulation of its gene product in the *hexR* mutant.

### Lack of an effect of HexR on protein secretion and hypersensitive response

**Figure 6:**
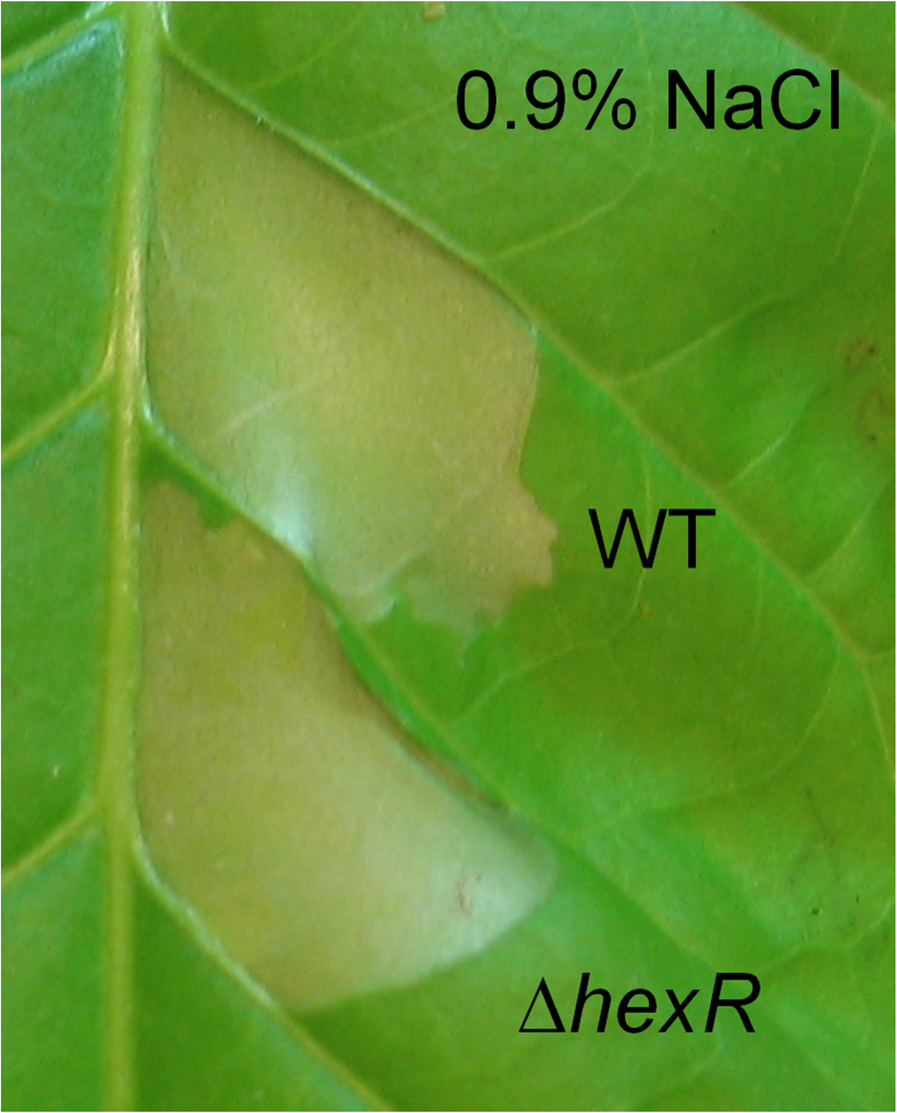
**Hypersensitive response assay on tobacco.** Typical hypersensitive response reactions elicited on tobacco plants as non-host defense responses by PG4180 wild type and its *hexR* mutant. Sterile 0.9% sodium chloride solution was used as a negative control.

**Figure 7:**
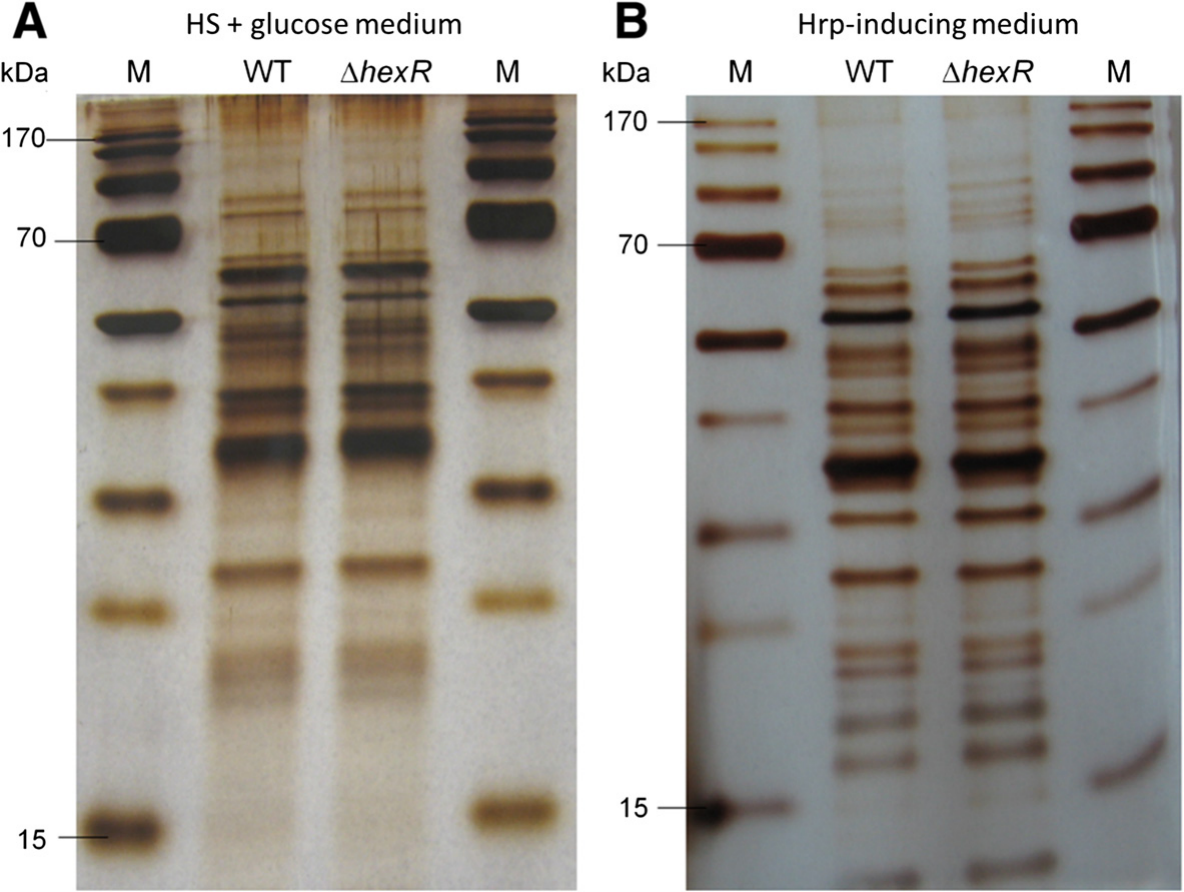
**Extra-cellular protein profiles for PG4180 wild type and its** ***hexR*** **mutant.** Bacterial cultures grown in **(A)** HS+ glucose medium or in **(B)** Hrp-inducing medium. Protein samples were separated by 12.5% SDS-PAGE and subsequently visualized via silver staining.

To further investigate the decreased *in planta* fitness of the *hexR* mutant of *P. syringae* as compared to its wild type, hypersensitive response (HR) assays were performed for both on the tobacco plant *Nicotiana tabacum* (Figure 6). The resulting HR after 24 hours on leaves inoculated with the mutant was indistinguishable from that induced by the wild type. In addition, extra-cellular protein profiles were determined for PG4180 wild type and its *hexR* mutant incubated in *hrp* gene-inducing IM medium or in HS+ glucose medium (Figure 7). After electrophoretic separation of extra-cellular proteins, nearly identical proteins profiles were observed for PG4180 wild type and its *hexR* mutant. In summary, these results indicated that a mutation of *hexR* does not influence the ability of *P. syringae* to induce an HR or alter its protein secretion pattern.

## Discussion

This study revealed that expression of genes encoding for an extra-cellular protein appear to be co-regulated with genes required for central hexose metabolism in a Gram-negative bacterium. Complementing previous studies on the global hexose metabolism repressor, HexR, in *P. putida* [21-24], our results suggested that involvement of HexR in regulation of *lsc* expression might be a selective adaptation of the plant pathogen, *P. syringae*, to its well-studied infection cycle [42,43]. Once Lsc is secreted, cellular resources needed for its synthesis such as amino acyl residues are not available for the cell anymore. Consequently, *P. syringae* might repress Lsc synthesis in coordination with hexose utilization when sufficient levels of intra-cellular glucose are available to balance the cell’s energy demands.

In close proximity and upstream of the translation start site (TSS) of *P. syringae lsc* genes, palindromic sequences were identified, which resemble HexR binding sites previously predicted for *P. putida* [22]. Repressors such as HexR were suggested to bind to inverted repeats that partially or fully overlap RNA-polymerase binding sites [44].

Sucrose is the most abundant plant storage sugar [45]. Bacterial *in planta* and *in vitro* growth analyses indicated that the substrate of Lsc, sucrose, and in consequence its enzymatic product, glucose, seem to be major nutrient sources for *P. syringae* during *in planta* growth. This is in line with previous findings reporting high molecular abundances of sucrose in bean plant’s apoplastic fluids [12]. Consequently, expression and secretion of Lsc might be a fitness factor for the *in planta* life of *P. syringae*. Along the course of the experiment, the total populations of PG4180 wild type and its *hexR* mutant varied but were not significantly different from each other. This variation reflects a feature that is usually observed during assessment of bacterial populations [45]. LL Kinkel, M Wilson and SE Lindow [46] showed that two leaves of the same plant species might vary by over 10-fold in their total epiphytic bacteria. Moreover, a study by SS Hirano and CD Upper [47] also demonstrated that regardless of the geographic area, plant species, or time scale tested, variations in population sizes of *P. syringae* are common. Many factors such as availability of nutrients, bacterial immigration [46], and ability of bacteria to tolerate environmental stresses on leaf surfaces [48] contribute to the observed variations.

A tendency is seen regarding the percentage of internalized bacteria in plants, although it is not significantly different. PG4180 wild type shows higher percentage of internalized bacteria as opposed to those inoculated with the *hexR* mutant. On days 3, 5, and 7 post inoculation, the wild type had 20, 23 and 12% more internalized bacteria, respectively, than the *hexR* mutant. The high local abundance of sugars, mainly sucrose and glucose, in the apoplast of leave tissue [49] could potentially be responsible for the weaker multiplication of the *hexR* mutant inside the plant tissue. Consequently, it is hypothesized that HexR might play an important role in survival of PG4180 inside soybean leaves. In turn, a higher percentage of external *hexR* mutant cells were surviving on the leaf surface although not significantly different (data not shown). Although our study did not provide direct evidence for this, an increase in levan formation by the *hexR* mutation might have protected the mutant from damage by UV light or desiccation leading to higher survival rates on the leaf surface.

**Figure 8:**
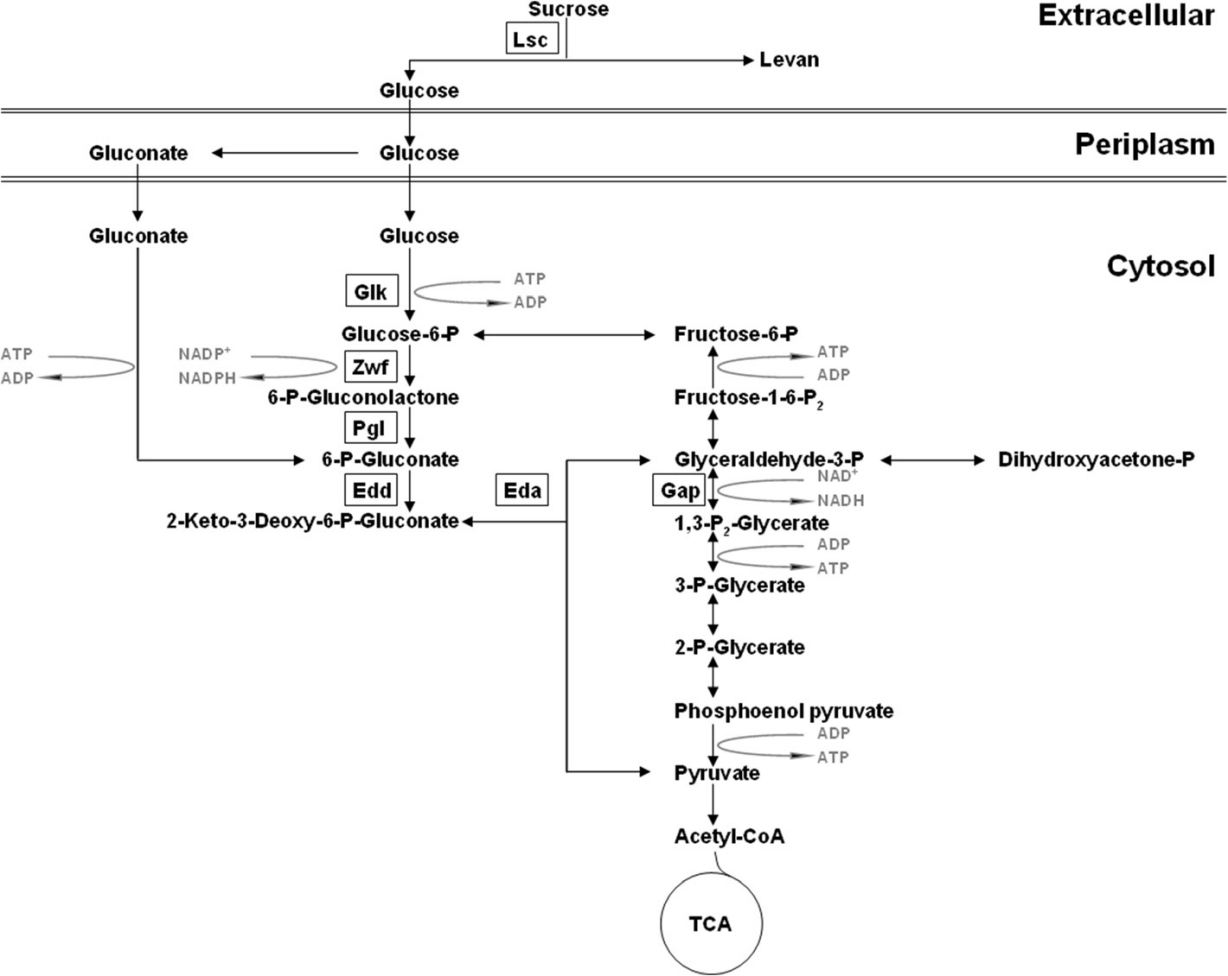
**Schematic presentation of putative sucrose utilization pathway in** ***P. syringae*** **PG4180.** Enzymes shown in blocks are presumed to be repressed by HexR in *P. putida* [22,23] or in *P. syringae* (present study). Lsc, levansucrase; Glk, glucose kinase; Zwf, glucose-6-phosphate dehydrogenase; Pgl, 6-phosphogluconolactonase; Edd, 6-phosphogluconate dehydratase; Eda, 2-keto-3-deoxy-6-phosphogluconate aldolase; Gap, glyceraldehyde 3-phosphate-dehydrogenase; TCA, tricarboxylic acid cycle.

Finally, one may hypothesize that production of the levan exopolymer would rather be a ‘shunt’ product during the release of glucose from sucrose which, in turn, could be the actual major function of Lsc in the sugar metabolic pathway (Figure 8). Genes encoding Lsc might be part of the HexR regulon of *P. syringae* in contrast to the situation in *P. putida* [23], which is neither phytopathogenic nor harboring any *lsc* genes. However, other bacterial species, which possess similar enzymes for cleavage of sucrose to obtain readily usable glucose, could show a similar HexR-mediated regulation. Therefore, it is suggestive to screen the most important oral cavity inhabiting bacterial species [50] as well as bacteria, which cause mucus formation in sucrose-based food manufacturing [51] or bio-fuel production [52] for presence of this regulatory linkage.

The use of arabinose as sole carbon source had no effect on the growth phenotype of the *hexR* mutant. This was not surprising since the assimilatory pathways of glucose and arabinose are independent. L-arabinose is converted to α–keto-glutarate in *Pseudomonas* which can directly be utilized in the Tricarboxylic acid cycle (TCA cycle) independent of HexR regulation [53,54].

Currently, there is no plausible explanation for the lack of significant growth of the *hexR* mutant when supplemented with glucose or sucrose as sole carbon source. However, one might speculate on an up-regulation of genes normally repressed by HexR such as *glk*, *zwf-1*, *pgl*, *edd*, *eda*, and *gap-1* as previously shown in *P. putida* [23]. The effect could be potentiated in *P. syringae* by an increased expression of *lsc*, whose gene product in turn provides even more glucose. Derepressed glucose consumption in the *hexR* mutant might cause a surplus production of NADPH, NADH, and ATP (Figure 8). In turn, this may lead to an imbalance of cellular redox homeostasis thus alleviating cellular ‘reductive stress’ or even inducing ‘energy spilling’, respectively.

In *E. coli*, the redox potential influences the synthesis of fermentation products, which are formed to recycle and reoxidize NADH [55]. When *E. coli* cells are aerobically challenged with high glucose concentrations, they undergo a so-called ‘acetate switch’, which decelerates growth [56]. A metabolic flux analysis of *E. coli* predicted that excess of carbon and energy might cause over-flow metabolism, which results in less efficient carbon utilization and decreased growth [57]. In *Streptococcus bovis*, excess ATP generation can cause ‘energy spilling’ by futile cycling of protons through the membrane, which leads to lesser biomass production [58,59]. Whether reductive stress or ‘energy spilling’ take place in a glucose-exposed *hexR* mutant of *P. syringae* remains to be analyzed in future studies.

Expression of *lsc* genes was higher in the *hexR* mutant as compared to the wild type during late logarithmic growth. This result is in accordance with previous results of micro-array analyses of glucose metabolic genes in *P. putida* KT2440 where genes *glk* and *zwf-1* showed a ~ two-fold increased expression while genes *pgl*, *edd*, *eda*, and *gap-1* exhibited a four- to six-fold increased expression in a respective *hexR* mutant [23]. Why *lsc* genes were only moderately up-regulated in the *hexR* mutant of *P. syringae* might be explained by the peripheral role of these genes in glucose metabolism.

The HR test is a classical assay to qualitatively show pathogenicity of a plant-associated microbe. The extracellular protein profiles and HR assay conducted with the wild type and the *hexR* mutant, respectively, suggested that HexR does not influence *P. syringae*’s ability to cause a HR on non-host plants. Furthermore, our results suggested that the secretion of *hrp*-associated proteins was not affected when *hexR* was mutated. It is therefore tempting to speculate that the reduced ability of the *hexR* mutant to survive inside of the plant is not due to altered *hrp* gene expression but is rather due to a distorted sugar metabolism.

## Conclusions

Data of this study prompt the question whether HexR-controlled genes such as *edd*, *eda*, *glk*, *pgl*, *zwf-1*, or *gap-1* [23] are indeed co-regulated with lsc genes in *P. syringae*. The current study revealed exciting options for an in-depth analysis of intra-cellular and extra-cellular hexose metabolism in the plant pathogen *P. syringae* and may allow us to better understand the potentially complex interplay of factors and parameters contributing to epiphytic or pathogenic behavior of this organism, respectively.

## Additional files

Additional file 1: Figure S1. Verification of *hexR* null mutant phenotype by PCR amplification. The total RNA was extracted by phenol chloroform method followed by cDNA generation. PCR amplification of *hexR* fragment on total cDNA and genomic DNA (gDNA) using *hexR* specific primers. The quality of total cDNA and genomic DNA were checked by performing PCR amplification of *lscB/C* gene which signified correct amplicon. H = *hexR* mutant, WT = PG4180 wild type. Additional file 2: Figure S2. Sequence alignment of a *P. syringae HexR* consensus sequence with that of *P. putida* KT2440 *HexR* [22]. Mismatched residues are marked in blue. Residues marked in red are the predicted DNA recognition (Q46, K49, E52, R57, R60) and effector recognition (S143, S187) residues of HexR, respectively [22]. Additional file 3: Figure S3. Multiple sequence alignment of HexR amino acid sequences from levan-producing *Pseudomonas syringae* strains using ClustalW [60]. PFAM comparison revealed two domains: Helix-turn-helix domain from residues 6–108 (PFAM PF01418) shown in pale grey and SIS domain from residues 128–256 (PFAM PF01380) shown in dark grey. Mismatched residues are marked in blue. Residues marked in red are the predicted DNA recognition (Q46, K49, E52, R57, R60) and effector recognition (S143, S187) residues of HexR, respectively [22].
