# Supplementary figures and images for: Expression of extra-cellular levansucrase in Pseudomonas syringae is controlled by the in planta fitness-promoting metabolic repressor HexR
Source: BMC Microbiol. 2015 Feb 26;15:48. doi: 10.1186/s12866-015-0349-0 (PMC4357207; doi:10.1186/s12866-015-0349-0)

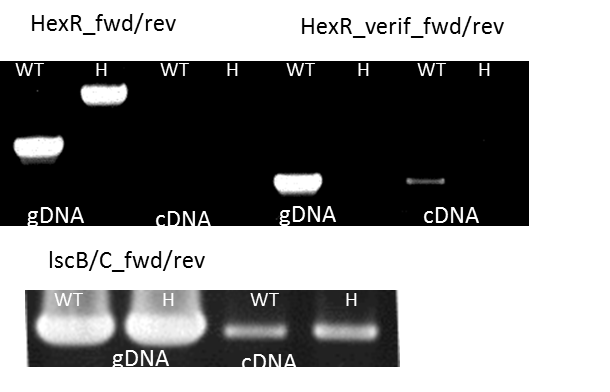

Supplement: Additional file 1: Figure S1. — Verification of hexR null mutant phenotype by PCR amplification. The total RNA was extracted by phenol chloroform method followed by cDNA generation. PCR amplification of hexR fragment on total cDNA and genomic DNA (gDNA) using hexR specific primers. The quality of total cDNA and genomic DNA were checked by performing PCR amplification of lscB/C gene which signified correct amplicon. H = hexR mutant, WT = PG4180 wild type. [file 12866_2015_349_MOESM1_ESM.tiff]

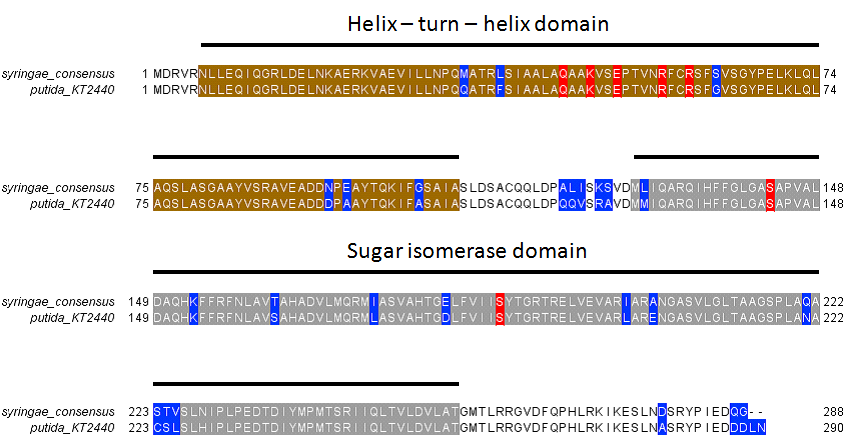

Supplement: Additional file 2: Figure S2. — Sequence alignment of a P. syringae HexR consensus sequence with that of P. putida KT2440 HexR [22]. Mismatched residues are marked in blue. Residues marked in red are the predicted DNA recognition (Q46, K49, E52, R57, R60) and effector recognition (S143, S187) residues of HexR, respectively [22]. [file 12866_2015_349_MOESM2_ESM.tiff]

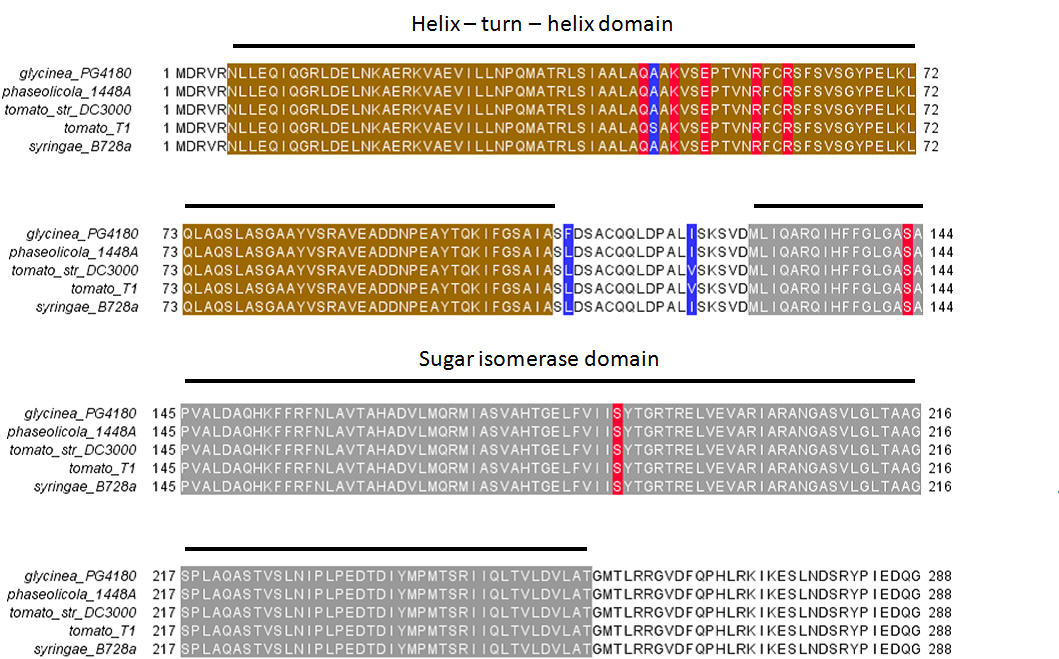

Supplement: Additional file 3: Figure S3. — Multiple sequence alignment of HexR amino acid sequences from levan-producing Pseudomonas syringae strains using ClustalW [60]. PFAM comparison revealed two domains: Helix-turn-helix domain from residues 6–108 (PFAM PF01418) shown in pale grey and SIS domain from residues 128–256 (PFAM PF01380) shown in dark grey. Mismatched residues are marked in blue. Residues marked in red are the predicted DNA recognition (Q46, K49, E52, R57, R60) and effector recognition (S143, S187) residues of HexR, respectively [22]. [file 12866_2015_349_MOESM3_ESM.tiff]
